# Supplementary material for: Optimizing automated sleep stage scoring of 5-s mini-epochs: a transfer learning study
Source: Sleep. 2025 Dec 12;49(4):zsaf393. doi: 10.1093/sleep/zsaf393 (PMC13089430; doi:10.1093/sleep/zsaf393)
Supplement: Article_2_Supplementary_material_081225_zsaf393 [file article_2_supplementary_material_081225_zsaf393.docx]

Optimizing automated sleep stage scoring of 5-second mini-epochs: a transfer learning study

Louise Frøstrup Follin^1,2,^*, Julie Anja Engelhard Christensen^1^, Janita Vevelstad^1^, Hilde T. Juvodden^1^, Rannveig Viste^1^, Berit Hjelde Hansen^1^, Mathias Perslev^3^, Tobias Kaufmann^4,5,6^, Alexander Neergaard Zahid ^7,8,#^, Stine Knudsen-Heier^1,2#^

^1^Norwegian Centre of Expertise for Neurodevelopmental Disorders and Hypersomnias (NevSom), Department of Rare Disorders, Oslo University Hospital, Norway

^2^Institute of Clinical Medicine, University of Oslo, Norway

^3^Danish Center for Sleep Medicine, Rigshospitalet, Denmark

^4^Centre for Precision Psychiatry, Institute of Clinical Medicine, University of Oslo, Norway

^5^Department of Psychiatry and Psychotherapy, University of Tübingen, Germany

^6^German Center for Mental Health (DZPG), Partner site Tübingen, Tübingen, Germany

^7^Department of Applied Mathematics and Computer Science, Technical University of Denmark, Kgs. Lyngby, Denmark

^8^WS Audiology, Lynge, Denmark

^#^These authors have contributed equally

*Corresponding author: Louise Frøstrup Follin, Norwegian Centre of Expertise for Neurodevelopmental Disorders and Hypersomnias (NevSom), Oslo University Hospital, Box 4956 Nydalen, 0424 Oslo, Norway. Email: [lofroe@ous-hf.no](mailto:lofroe@ous-hf.no)

## Table of contents

Manual for scoring 5-second mini-epochs Page 2

Additional mini-epoch scoring notes Page 5

Figure S1 Page 6

Table S1 Page 7

Figure S2 Page 8

Table S2 Page 9

Table S3 Page 10

Table S4 Page 11

Table S5 Page 12

## Manual for scoring 5-second mini-epochs

### General Scoring Rules

1. **Scrolling**: It is allowed to scroll back and forth within a 20-minute segment.
2. **Independent Scoring**: Each 5-second mini-epoch is scored as independently as possible; however, it is allowed to rely on context from previous or subsequent mini-epochs.
3. **Characteristics**: If one characteristic from a stage (listed below) is present in a given mini-epoch, that stage should be scored.
4. **Majority Rule**: If two or more stages are present in the same mini-epoch, score the stage that fills out the majority of the mini-epoch.
5. **No characteristics**: If no characteristics are present, there is the opportunity to mark this, however the context of surrounding mini-epoch along with pattern recognition should be used to decide which sleep stage it most certain is. This opportunity does not apply to wake and N3.
6. **Tonus**: Low tonus is not a specific characteristic as tonus can be low throughout all night. If tonus is used for pattern recognition, the context of the surrounding mini-epoch should be used to decide sleep stage.

### Stage characteristics

**Wake (W)**

- ≥50% Alpha activity.

**N1:**

- Slow eye movements.
- Vertex sharp waves.
- Note: If no slow eye movements or vertex sharp waves are seen in a given mini-epoch and no other characteristics suggesting other sleep stages are present and surrounding mini-epochs are scored as N1 and/or the background EEG resembles N1 or does not suggest another stage score “N1 without characteristics”.

**N2:**

- Sleep spindles.
- K-complexes.
- Note: If no sleep spindle or K-complex are seen in a given mini-epoch, and no other characteristics suggesting other sleep stages are present and surrounding mini-epochs are scored as N2 and/or the background EEG resembles N2 or does not suggest another stage score “N2 without characteristics”.

**N3:**

- High-amplitude, low-frequency delta waves (SWS).

**REM:**

- Rapid eye movements.
- Note: A rapid eye movement must be present in the mini-epoch for “REM” to be scored, otherwise if no other characteristics suggesting other sleep stages are present, and the surrounding mini-epochs are scored as REM, and/or the background EEG resembles REM or does not suggest another stage; score “REM without characteristics”.
- Note: If there is more than 50% alpha activity in the mini-epoch but no eye movements, however, it looks more like REM sleep than wake; “REM with alpha” should be scored.
- Note: If there is more than 50% alpha activity in the mini-epoch but eye movements and it looks more like REM sleep than wake; “REM” should be scored.

### Protocol

**Step 1:** The human scorer has 10 minutes to investigate a given full PSG in DOMINO.

**Step 2:** The human scorer scores mini-epochs of the first 20-minute segment from a given PSG in MATLAB following the rules above.

**Step 3:** The human scorer scores mini-epochs of the second 20-minute segment from a given PSG in MATLAB following the rules above.

The two segments from each PSG are in random order, meaning the first segment may originate from a later time in the night than the second segment. These three steps are repeated for all PSGs.

## Additional mini-epoch scoring notes

Although the scoring manual allowed for the option “REM with alpha” (defined as >50% alpha activity without eye movements, but the overall mini-epoch resembling REM more than wake, see above), this category was not included in the analyses presented in the manuscript as it was rarely occurring in actual dataset, accounting for only 0.095% of all human-scored mini-epochs


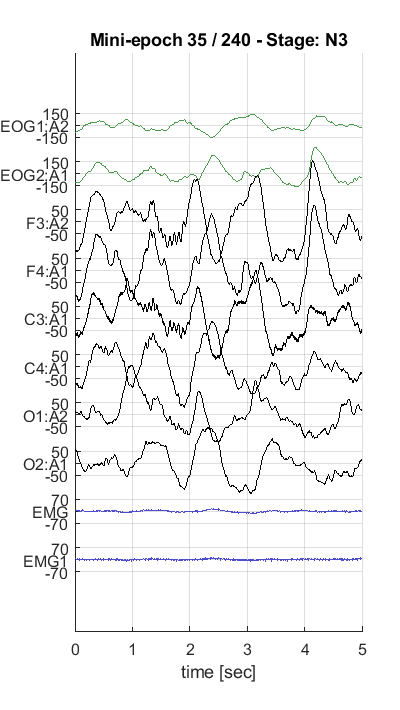


Figure S1: MATLAB 2020b based scoring interface designed to mimic the SOMNOmedics DOMINO software layout. The example shows a 5-second mini-epoch with EEG, EOG, and EMG channels displayed for human scoring. The sleep stage label (here: N3) is printed only after the human scorer has assigned the stage.

| Dataset | Total number of participants (n=100) | NT1 patients  (n=38) | Siblings  (n=62) |
| --- | --- | --- | --- |
| Training | 80 | 30 | 50 |
| Validation | 10 | 4 | 6 |
| Testing | 10 | 4 | 6 |

*Table S1: Numbers of patients and siblings in the training, validation and test sets.*


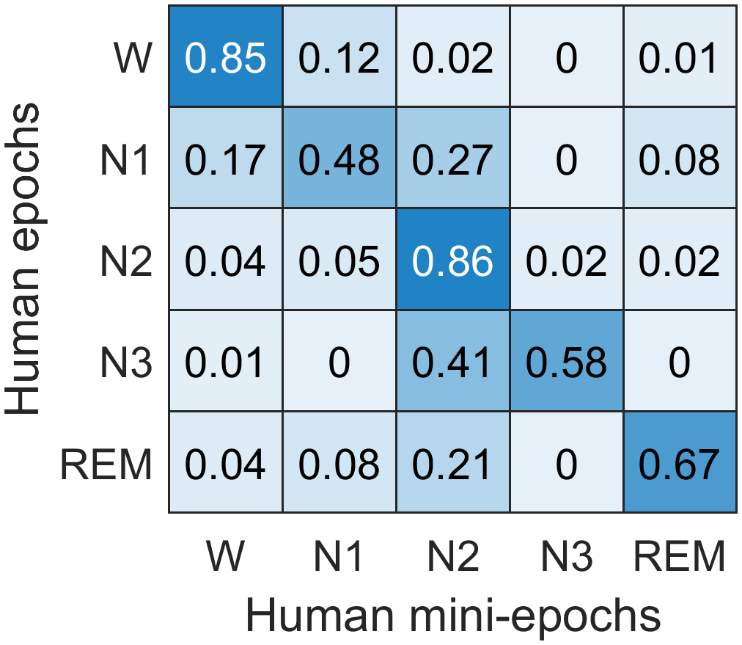


Figure S2: The figure is based on 100 PSGs, each contributing two 20-minute PSG segments. Recall weighted, row-wise normalized confusion matrix between human-scored epochs (rows) and human-scored mini-epochs (columns). Diagonal values represent agreement for each sleep stage. Off-diagonal values show misclassification patterns (e.g., row 3, column 2 indicates that 5% of the mini-epochs coming from N2 epochs were scored as N1 in the human-scored mini-epochs).

*Table S2: Comparison of F1-scores before and after training of U-Sleep (original versus optimized U-Sleep model). The reported is: Median difference and bootstrapped 95% CI, effect size* $r=Z/\sqrt{n}$ *and p-value found via the Wilcoxon signed-rank.*

|  | Median difference (95% CI) | Non-zero pairs (N) | Effect size (r) | P-value |
| --- | --- | --- | --- | --- |
| Wake | 0.11 (0.06; 0.29) | 10 | 0.69 | 0.028 |
| N1 | 0.15 (0; 0.45) | 8 | 0.48 | 0.173 |
| N2 | 0.07 (0.01; 0.15) | 9 | 0.61 | 0.051 |
| N3 | 0.01 (0; 0.09) | 8 | 0.75 | 0.068 |
| REM | 0.01 (-0.04; 0.05) | 6 | 0.30 | 0.345 |
| Overall | 0.06 (0.03; 0.11) | 10 | 0.66 | 0.037 |

*Table S3: Mean F1-scores from the 5-fold cross-validation.*

|  | Wake | N1 | N2 | N3 | REM | Overall |
| --- | --- | --- | --- | --- | --- | --- |
| Fold 1 | 0.62 | 0.43 | 0.84 | 0.59 | 0.79 | 0.83 |
| Fold 2 | 0.63 | 0.25 | 0.74 | 0.50 | 0.61 | 0.76 |
| Fold 3 | 0.68 | 0.39 | 0.80 | 0.61 | 0.64 | 0.80 |
| Fold 4 | 0.65 | 0.40 | 0.83 | 0.58 | 0.61 | 0.77 |
| Fold 5 | 0.65 | 0.47 | 0.80 | 0.55 | 0.69 | 0.80 |
| Mean | 0.64±0.03 | 0.39±0.08 | 0.80±0.04 | 0.57±0.04 | 0.67±0.08 | 0.79±0.03 |

*Table S4: Mean F1-scores with bootstrapped 95% confidence intervals for each sleep stage and overall, stratified by group. Results are shown for the original U-Sleep model (before transfer learning) and the optimized U-Sleep model (after transfer learning), separately for siblings and patients with narcolepsy type 1 (NT1).*

|  | W | N1 | N2 | N3 | REM | Overall |
| --- | --- | --- | --- | --- | --- | --- |
| Original U-Sleep model | | | | | | |
| Siblings | 0.51  (0.23–0.71) | 0.22  (0.00–0.50) | 0.71  (0.55–0.82) | 0.78  (0.50–0.93) | 0.67  (0.59–0.82) | 0.74  (0.65–0.81) |
| NT1 | 0.53  (0.27–0.65) | 0.27  (0.11–0.38) | 0.80  (0.69–0.89) | 0.65  (0.00–0.96) | 0.80  (0.51–0.96) | 0.75  (0.63–0.87) |
| Optimized U-Sleep model | | | | | | |
| Siblings | 0.61  (0.27–0.91) | 0.26  (0.04–0.55) | 0.82  (0.68–0.92) | 0.87  (0.63–0.97) | 0.69  (0.61–0.86) | 0.83  (0.74–0.90) |
| NT1 | 0.62  (0.36–0.76) | 0.50  (0.32–0.65) | 0.82  (0.64–0.90) | 0.67  (0.00–0.96) | 0.75  (0.51–0.88) | 0.78  (0.70–0.87) |

*Table S5: Sleep–wake transitions per minute in patients with narcolepsy type 1 (NT1) and siblings based on human-scored 30-second epochs and 5-second mini-epochs scored by the human, original U-Sleep and optimized U-Sleep model. P-values are from linear mixed-effects models adjusted for age, sex, and family ID. Outcomes were square-root transformed to meet model assumptions. Effect sizes were calculated as the estimated fixed effect for group divided by the residual standard deviation from the mixed model, representing standardized mean differences on the transformed scale.*

|  | Siblings, mean ± std | NT1, mean ± std | Effect size (Cohen’s *d*) | p-value |
| --- | --- | --- | --- | --- |
| Human-scored epochs, sleep-wake transitions/minute | 0.041 ± 0.053 | 0.070 ± 0.075 | 0.46 | 0.040 |
| Human-scored mini-epochs, sleep-wake transitions/minute | 0.388 ± 0.426 | 0.546 ± 0.460 | 0.55 | 0.014 |
| Original U-Sleep model, sleep-wake transitions/minute | 0.269 ± 0.184 | 0.467 ± 0.308 | 0.80 | 0.0002 |
| Optimized U-Sleep model, sleep-wake transitions/minute | 0.327 ± 0.342 | 0.384 ± 0.322 | 0.75 | 0.0004 |
